# Viburnum Prunifolium

**Published:** 1882-02-20

**Authors:** Thomas F. Houston

**Affiliations:** Clarkesville, Ga.


### Southern Medical Record:

EDITORS:

T. S. POWELL, M.D. W. T. GOLDSMITH, M.D. R. C. WORD, M.D.

*R. C. WORD, M.D., Managing Editor.*

☞All Communications and Letters on Business connected with the RECORD must be addressed to the Managing Editor.

VOL. XII. ATLANTA. GA., FEBRUARY 20, 1882. No. 2.

### ORIGINAL AND SELECTED ARTICLES.

### VIBURNUM PRUNIFOLIUM.

BY THOMAS F. HOUSTON, M. D., OF CLARKESVILLE, GA.

There is a trite old saying, "When doctors differ then the devil's to pay," but in spite of this warning, I feel constrained to take issue with Dr. A. G. Smythe on account of an article upon this subject, published in the RECORD for December. The writer utterly annihilates this valuable drug, saying that no remedy had so utterly failed him as viburnum, during a practice of forty-five years. While I would not, for one moment, question the Doctor's veracity, I am at a loss to understand these results, so diametrically opposite to my experience in its use. And I must attribute his want of success to the inferiority of the article, or the smallness of the dose. Being a new and comparatively little used remedy, there is not the same care taken in its collection and manufacture as there is of the standard drugs. In regard to the dose, the various works give the dose of the fluid extract (the only form in which the drug should be used) as one drachm. I rarely give less than two, and repeat this every fifteen minutes until I get the desired effect, if I have to give a fluid ounce. In fact, my method of using the drug is to give to my pregnant patients, if there is the slightest tendency to abortion, a bottle of the fluid extract, and direct them to take from one to four drachms every time they feel any pelvic uneasiness, proportioning the dose to the amount of the disturbance, and to assume the recumbent posture. If the patient has ever miscarried, I direct her to take one drachm three times a day during the week in each month that the menses would be present, did not the pregnancy exist. I could cite many cases in which this treatment succeeded perfectly—in one, the lady went to full time, after having miscarried seven times.

Of course I do not claim that black haw will cause the retention of a dead fœtus, or remove the reflex irritation caused by an ulcer or a laceration of the cervix, or other mechanical or pathological reason for this attempt of the uterus to expel its contents. But in the condition known as irritable uterus, due entirely to nervous action, caused by over exertion, fright, mental worry, an abortive tendency, etc., if the dose is sufficiently large and given early enough in the attack, viburnum will prove itself worthy of the confidence that I ask for it. In cases in which worry or mental excitement or grief is the exciting cause, the bromides, tincture of valerian, or valerianate of ammonia, is a valuable addition. Though I have no ambition to "excel my predecessors in sounding the virtues, claims and praises of viburnum prunifolium in the prevention and arrest of abortion, miscarriage and all the concomitant troubles attending that condition," still I must say that, in my humble judgment, if it is used in the class of cases given, and with sufficient promptness, it is as much a specific for abortion as quinine for malaria, and aconite for inflammatory fever.

Speaking of quinine, I give it in full doses to pregnant women *without hesitancy*, simply combining a corresponding dose of black haw with it. Again, there is nothing better for after-pains, and this very evening, since I commenced to write this article, I was summoned to see Mrs. ——, suffering with neuralgic dysmenorrhœa which was relieved in 15 minutes by ʒii viburnum and 5 grs. croton chloral hydrate. In ordinary cases the croton chloral is not needed, but if there is much nervousness it is well to combine it.

I trust Dr. Smythe will pardon my differing so strongly with him, but it is in the hope that it will call out the opinions of others who have tested this drug, and who are more competent to defend it than I am.

---

FLUID extracts of digitalis and colchicum are said by Dr. Squibb to be more eligible preparations than the corresponding tinctures. They are eight times as strong, and cost only half as much.—[Louisville Medical News.

